# Supplementary material for: Antibody Tests in Detecting SARS-CoV-2 Infection: A Meta-Analysis
Source: Diagnostics (Basel). 2020 May 19;10(5):319. doi: 10.3390/diagnostics10050319 (PMC7278002; doi:10.3390/diagnostics10050319)

Supplementary Table 1: The QUADAS tool

Item		Yes	No	Unclear
1.	Was the spectrum of patients representative of the patients who will receive the test in practice?	(38)	(-)	(-)
2.	Were selection criteria clearly described?	(38)	(-)	(-)
3.	Is the reference standard likely to correctly classify the target condition?	(38)	(-)	(-)
4.	Is the time period between reference standard and index test short enough to be reasonably sure that the target condition did not change between the two tests?	(31)	(-)	(7)
5.	Did the whole sample or a random selection of the sample, receive verification using a reference standard of diagnosis?	(38)	(-)	(-)
6.	Did patients receive the same reference standard regardless of the index test result?	(36)	(2)	(-)
7.	Was the reference standard independent of the index test (i.e., the index test did not form part of the reference standard)?	(38)	(-)	(-)
8.	Was the execution of the index test described in sufficient detail to permit replication of the test?	(38)	(-)	(-)
9.	Was the execution of the reference standard described in sufficient detail to permit its replication?	(38)	(-)	(-)
10.	Were the index test results interpreted without knowledge of the results of the reference standard?	(36)	(-)	(2)
11.	Were the reference standard results interpreted without knowledge of the results of the index test?	(38)	(-)	(-)
12.	Were the same clinical data available when test results were interpreted as would be available when the test is used in practice?	(38)	(-)	(-)
13.	Were uninterpretable/ intermediate test results reported?	(38)	(-)	(-)
14.	Were withdrawals from the study explained?	(38)	(-)	(-)

Whiting P, Rutjes AW, Reitsma JB, et al. The development of QUADAS: a tool for the quality assessment of studies of diagnostic accuracy included in systematic reviews. BMC Med Res Methodol 2003;3:25

Supplementary Table 2: The Quadas Tool 2

	Bias				Applicability		
	PATIENT SELECTION	INDEX TEST	REFERENCE STANDARD	FLOW AND TIMING	PATIENT SELECTION	INDEX TEST	REFERENCE STANDARD
Study 1	Low	Low	Low	Low	Low	Low	Low
Study 2	Low	Low	Low	Low	Low	Low	Low
Study 3	Low	High	Low	Unclear	Low	High	Low
Study 4	Low	Low	Low	Low	Low	Low	Low
Study 5	Low	Low	Low	Low	Low	Low	Low
Study 6	Low	Low	Low	Low	Low	Low	Low
Study 7	Low	Low	Low	Low	Low	Low	Low
Study 8	Low	Low	Low	Low	Low	Low	Low
Study 9	Low	Low	Low	Low	Low	Low	Low
Study 10	Low	Unclear	Low	High	Low	Unclear	Low
Study 11	Low	Unclear	Low	High	Low	Unclear	Low
Study 12	Low	Low	Low	Low	Low	Low	Low
Study 13	Low	Low	Low	Low	Low	Low	Low
Study 14	Low	Low	Low	Low	Low	Low	Low
Study 15	Low	Low	Low	Low	Low	Low	Low
Study 16	Low	High	Low	Low	Low	High	Low
Study 17	Low	Low	Low	Low	Low	Low	Low
Study 18	Low	High	Low	Low	Low	High	Low
Study 19	Low	Low	Low	Low	Low	Low	Low
Study 20	Low	Low	Low	Low	Low	Low	Low
Study 21	Low	Low	Low	Low	Low	Low	Low
Study 22	Low	Low	Low	Low	Low	Low	Low
Study 23	Low	Low	Low	Low	Low	Low	Low
Study 24	Low	Low	Low	Low	Low	Low	Low
Study 25	Low	Low	Low	Low	Low	Low	Low
Study 26	Low	High	Low	Low	Low	High	Low
Study 27	Low	High	Low	Low	Low	High	Low
Study 28	Low	Low	Low	Low	Low	Low	Low
Study 29	Low	Low	Low	Low	Low	Low	Low
Study 30	Low	Low	Low	Low	Low	Low	Low
Study 31	Low	Low	Low	Low	Low	Low	Low
Study 32	Low	Low	Low	Low	Low	Low	Low
Study 33	Low	High	Low	Low	Low	High	Low
Study 34	Low	Low	Low	Low	Low	Low	Low
Study 35	Low	Unclear	Low	Low	Low	Unclear	Low
Study 36	Low	Low	Low	Low	Low	Low	Low
Study 37	Low	Low	Low	Low	Low	Low	Low
Study 38	Low	High	Low	Low	Low	High	Low

Supplementary Figure 1: The Quadas Tool 2 graph

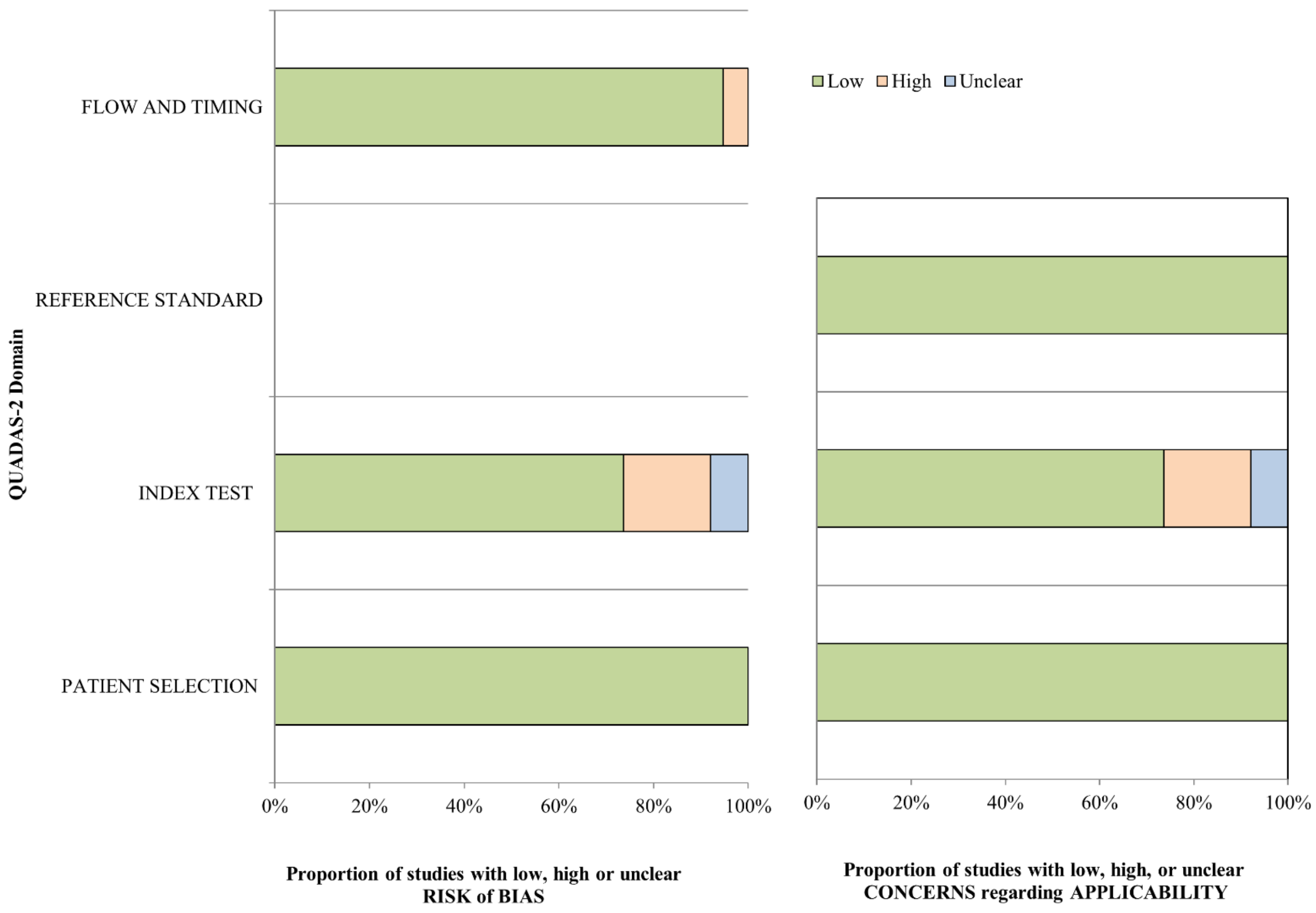

Supplement: Supplementary file 1 [file diagnostics-10-00319-s001.pdf]
